# Immunohistochemical and molecular profiles of heterogeneous components of metaplastic breast cancer: a squamous cell carcinomatous component was distinct from a spindle cell carcinomatous component

**DOI:** 10.1007/s12672-024-00950-0

**Published:** 2024-04-02

**Authors:** Takahiro Suzuki, Yoko Nakanishi, Tomoyuki Tanino, Haruna Nishimaki-Watanabe, Hiroko Kobayashi, Sumie Ohni, Xiaoyan Tang, Kenichi Hakamada, Shinobu Masuda

**Affiliations:** 1 Division of Oncologic Pathology, Department of Pathology and Microbiology, Nihon University School of Medicine, 30-1 Oyaguchi Kami-cho, Itabashi-ku, Tokyo, Japan; 2 Department of Gastroenterological Surgery, Hirosaki University Graduate School of Medicine, 5 Zaifu-cho, Hirosaki, Aomori Japan

**Keywords:** Metaplastic breast carcinoma, Squamous cell carcinoma, Spindle cell carcinoma, Heterogeneity, Phosphatase, Tensin homologue

## Abstract

Metaplastic breast carcinoma (MBC), a category of breast cancer, includes different histological types, which are occasionally mixed and heterogeneous. Considering the heterogeneity of cancer cells in a tumour mass has become highly significant, not only from a biological aspect but also for clinical management of recurrence. This study aimed to analyse the immunohistochemical and molecular profiles of each MBC component of a tumour mass. Twenty-five MBC tumours were histologically evaluated, and the most frequent MBC component (c) was squamous cell carcinoma (SCC), followed by spindle cell carcinoma (SpCC). A total of 69 components of MBC and non-MBC in formalin-fixed paraffin-embedded sections were examined for 7 markers by immunohistochemistry. SCC(c) were significantly PTEN negative and CK14 positive, and SpCC(c) were significantly E-cadherin negative and vimentin positive. Multivariate analyses revealed that immunohistochemical profiles of normal/intraductal (IC)(c), no special type (NST)(c), and MBC(c) differed; moreover, SCC(c) and SpCC(c) were distinctly grouped. *PTEN* gene mutation was detected only in SCC(c) (2/7), but not in SpCC(c). Next-generation sequence analyses for 2 cases with tumours containing SCC(c) demonstrated that *PTEN* gene mutation increased progressively from IC(c) to NST(c) to SCC(c). In conclusion, the immunohistochemical and molecular profiles of the SCC(c) of MBC are distinct from those of the SpCC(c).

**Supplementary Information:**

The online version contains supplementary material available at 10.1007/s12672-024-00950-0.

## Introduction

Metaplastic breast carcinoma (MBC) is a pathological category of breast cancer with histology that is characterized by dedifferentiation and metaplastic change deviated from ductal epithelial cells; MBC accounts for 0.2–1% of all invasive breast carcinomas [1]. Overall, the outcomes of patients with MBC are worse than those of patients with non-MBC [2, 3]. In particular, the spindle cell carcinoma (SpCC) subtype is associated with poorer disease-free survival (DFS) and overall survival (OS) than other subtypes [3–5]. MBC is generally oestrogen receptor (ER) negative, progesterone receptor (PgR) negative and human epidermal growth factor receptor 2 (HER2) negative, categorized as triple-negative breast carcinoma (TNBC) [2, 3]. Comprehensive gene expression analyses have revealed that the composition of intrinsic subtypes of MBC differ from those TNBC of no special type (NST) [6, 7]. Recent comprehensive genetic analyses for MBC have revealed abnormalities in *TP53* (56–75%), the phosphatidylinositol-3 kinase catalytic subunit/AKT pathway (*PIK3CA*; 23–48%, *PTEN*; 11–25%), the WNT pathway (*FAT1*; 11%), and cell cycle-related genes (*CCNE1, CCND3*, and *CDKN2A/B*) [8–12]. Although the frequency of *TP53* gene mutation does not differ between MBC and TNBC [10], somatic mutations in *PIK3CA, PTEN* [9, 10], and *NF1* [9] occur more frequently in MBC than in TNBC of NST [10]. Based on these results, MBC has unique phenotypic and genetic characteristics.

Subtypes of MBC include fibromatosis-like metaplastic carcinoma, squamous cell carcinoma (SCC), spindle cell carcinoma (SpCC), metaplastic carcinoma with heterologous mesenchymal differentiation (sarcomatoid/pleomorphic carcinoma (PLM) or matrix-producing carcinoma (MPC), potentially with chondroid/cartilaginous (CAR) differentiation and osseous (OSS) differentiation) [1]. The prevalence of these histological types is 17–52% for SCC, 13–34% for SpCC, 9–29% for MPC, 10.4% for CAR, 4–7% for fibromatosis-like carcinoma, and 3.0% for OSS [2, 3]. Nevertheless, MBC tumours actually contain heterogeneous histologies, even when diagnosed as one histological type of MBC.

Understanding the heterogeneity of cancer cells in a tumour mass has become increasingly important. The morphological heterogeneity observed by histology has attracted the attention of pathologists from a taxonomic point of view, in the past. At the present time, we can understand the process of carcinogenesis via integrated information of phenotypic and genetic knowledge for cancer cells. One such example is mixed ductal and lobular breast cancer. Although ductal carcinoma and lobular carcinoma are classified as different histological types, recent investigations have revealed that intimate mixtures of ductal and lobular carcinoma cells might share genetic alterations and originate from common ancestor cells [13, 14]. Such biological and pathological understanding of heterogeneity is also critical for adequate treatment and clinical management for recurrent lesions, because a tumour mass containing heterogeneous components is associated with increased risk of remnant and recurrent cancer cells resistant to initial treatments.

MBC tumours comprise rather heterogeneous cancer cells; however, from phenotypic and genetic perspectives, it has not been fully clarified whether cancer cells with a deviated “metaplastic” phenotype arises from carcinoma cells of no special type with acquired additional genetic alterations, or de novo from non-neoplastic epithelial cells. Therefore, we aimed to clarify the lineage among heterogeneous components of an MBC tumour mass by analysing immunohistochemical profiles and gene mutations focusing on each histological component.

## Materials and methods

### Patients

The patients enrolled in this study were selected from the medical records of Nihon University Itabashi Hospital. Inclusion criteria were patients with primary breast cancer diagnosed as MBC who underwent mastectomy in our hospital from 2000 to 2018. Exclusion criteria were receipt of any treatment before surgery. Twenty-five cases were selected, and the clinical data collected were age and presence of recurrence and metastasis; pathological data collected were tumour size, nuclear grade, pathological TNM and stage, vessel invasion and subtype (Supplementary Table 1). This study was conducted in accordance with the Declaration of Helsinki of 1975 and approved by the Institutional Review Board of Nihon University Itabashi Hospital (Approval number: RK-160712-11). This study was a retrospective, observational study, and the consent of patients was waived by the IRB, with an opportunity to decline participation.

### Histopathological examination

**Fig. 1:**
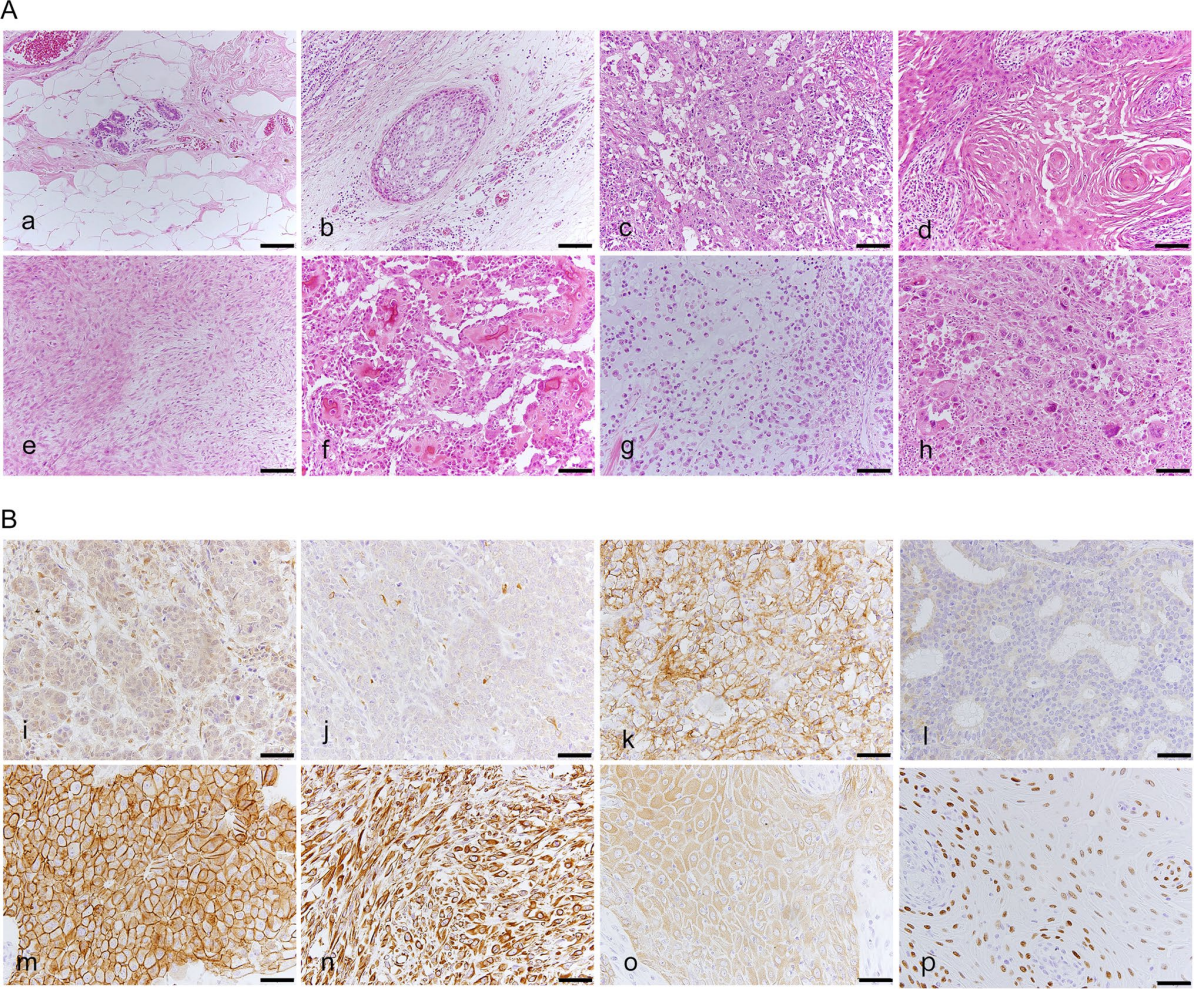
Representative histological features of evaluated components in MBC and immunohistochemical findings. **a** Representative histological features of the eight components of MBC (haematoxylin and eosin (HE) staining). **a** Normal breast tissue (normal(c)), **b** intraductal (IC(c)), **c** invasive ductal carcinoma of no special type (NST(c)), d: squamous cell carcinoma (SCC(c)), **e** spindle cell carcinoma (SpCC(c)), **f** carcinoma with cartilaginous/osseous differentiation (CAR/OSS(c)), **g** matrix-producing carcinoma (MPC(c)), **h** pleomorphic carcinoma (PLM(c)). Scale bars: 100 µm. **b** Representative immunohistochemical findings of the following proteins stained according to protocols described in the Materials and Methods. **i** phosphatase and tensin homologue (PTEN), **j** liver kinase B1 (LKB1), **k** cluster of differentiation 44 (CD44), **l** transforming growth factor-β receptor 1 (TGFBR1), **m** E-cadherin, **n** vimentin, **o** cytokeratin 14 (CK14), and **p** p40, Scale bars: 50 µm

The resected breast cancer tissues were fixed in 10% neutral buffered formalin and processed appropriately according to routine practice; 4 µm formalin-fixed paraffin-embedded (FFPE) sections were stained with haematoxylin and eosin (HE), and examined by 2 certified pathologists and additional certified pathologist. Histopathological diagnosis was performed according to World Health Organization (WHO) Classification of Tumours of the Breast [15]. Based on medical records, no patients had pathological diagnoses of low-grade adenosquamous carcinoma or fibromatosis-like metaplastic carcinoma. Five cases of SpCC, 2 cases of SCC, 4 cases of metaplastic carcinoma with heterologous mesenchymal differentiation and 14 cases of mixed metaplastic carcinoma were included in this study. To examine how metaplastic components develop their phenotypic characteristics, we evaluated heterogeneous components individually, e.g., a no special type (NST) component (c) in mixed metaplastic carcinoma, an intraductal component (IC)(c), and a normal breast tissue (normal)(c), in addition to MBC(c)(SCC(c), SpCC(c), OSS/CAR(c), MPC(c), and PLM(c)) (Fig. 1a–h) (Supplementary Table 2). All immunohistochemical evaluations and next-generation sequencing (NGS) analyses were performed individually for these histological components.

### Immunohistochemistry (IHC)

Four-micron-thick FFPE sections were used for immunohistochemistry (IHC) with antigen retrieval under the appropriate condition for each antibody (Supplementary Table 3). Primary antibodies against PTEN, liver kinase B1 (LKB1), E-cadherin, vimentin, CD44, cytokeratin 14 (CK14), p40, and transforming growth factor-beta receptor 1 (TGFBR1) were incubated at 4 ℃ overnight. The slides were incubated with Simple Stain MAX-PO (Multi) (Nichirei Bioscience Inc., Tokyo, Japan) for 30 min at RT, then incubated with 3,3′-diaminobenzidine tetrahydrochloride (DAB) for 10 min at RT for visualization, and counterstained with haematoxylin.

Immunohistochemical findings were evaluated by certified breast pathologists and classified as positive when > 5% of the tumour cells showed staining. PTEN, LKB1, TGFBR1, vimentin, and CK14 were detected in the cytoplasm; CD44 and E-cadherin were detected at the cell membrane; and p40 was detected in the nucleus (Fig. 1i–p). IHC for p40 was used only to confirm the squamous phenotype indicated by HE staining.

The positive controls used were normal breast tissue for PTEN; colon cancer tissue for LKB1 and TGFBR1; skin tissue for E-cadherin and CK14; adipose tissue for vimentin; and cutaneous squamous cell carcinoma for CD44 and p40. A negative control was performed by omitting the primary antibodies and using control antibodies (mouse IgG and rabbit IgG, Vector Laboratories, Inc., CA, U.S.A.)

### *PTEN* gene mutation analysis

*PTEN* gene mutations were examined using whole slides of MBC tissues. DNA was extracted from the tissues using the QIAamp DNA FFPE Tissue kit (QIAGEN, Hilden, Germany) according to the manufacturer’s instructions. The DNA concentration was assessed using a NanoDrop One instrument (Thermo Fisher Scientific, Waltham, MA, USA). Twelve samples with sufficient DNA (> 10 ng/μl) for analysis were selected for further examination.

Polymerase chain reaction (PCR) was performed with AmpliTaq Gold® 360 Master Mix (Thermo Fisher Scientific) and specific primers (Supplementary Table 4). The PCR conditions were 95 °C for 10 min, followed by 50 cycles of 95 °C for 30 s, 56 °C for 1 min, and 72 °C for 1 min. PCR was performed using a thermal cycler (PC808; ASTEC Co., Ltd., Fukuoka, Japan). The PCR products were confirmed by 2% agarose gel electrophoresis and purified using Illustra ExoProStar (GE Healthcare UK, Ltd., Buckinghamshire, UK). Sequencing reactions were performed with BigDye® Terminator v3.1 Cycle Sequencing Kit (Thermo Fisher Scientific) and specific PCR primers (Supplemental Table 4) with an ABI 3730xl sequencer (Thermo Fisher Scientific). Sequence similarities were analysed in silico using BLAST searches (http://blast.ncbi.nlm.nih.gov/Blast.cgi).

### Next-generation sequencing analysis

We analysed genetic changes in MBC, including SCC(c), of 2 cases (case no. 16, NST(c) and SCC(c); case no. 25, IC(c), NST(c) and SCC(c)) using NGS. Eight-micron-thick FFPE sections were cut and mounted onto glass slides coated with membrane film for laser microdissection. After deparaffinization, the sections were stained with toluidine blue and dried at RT. Each target component was dissected and collected using the PALM® MicroBeam III-N system (Carl Zeiss Microscopy, Munich, Germany). Genomic DNA was extracted using the QIAamp DNA FFPE Tissue kit (QIAGEN) according to the manufacturer’s instructions. Initial quality control checks of the FFPE DNA were performed using 1% agarose gel electrophoresis and Qubit dsDNA HS Assay Kit with a Qubit 2.0 fluorometer (Thermo Fisher Scientific). Amplicon libraries were prepared using Ion AmpliSeq™ Library Kit 2.0 (Thermo Fisher Scientific) with Ion AmpliSeq™ Comprehensive Cancer Panel (Thermo Fisher Scientific), which covers all exons of 409 cancer and cancer-related genes, according to the manufacturer’s instructions. Library quality was assessed using a 2100 Bioanalyzer (Agilent Technologies, Santa Clara, CA, USA); the libraries were quantified using Ion Library TaqMan® Quantitation Kit and a LightCycler® 480 Instrument II (Roche, Rotkreuz, Switzerland). Sequencing was performed with Ion Proton™ (Thermo Fisher Scientific) using PI Chip (Thermo Fisher Scientific). The sequencing data were analysed with Torrent Suite Software (Ver5.0.4; Thermo Fisher Scientific). Variants were filtered according to the following criteria: total allele coverage greater than 100 and variant allele frequency (VAF) greater than 4%. Germline mutations confirmed in the ClinVar database (https://www.ncbi.nlm.nih.gov/clinvar/) and single-nucleotide variants confirmed in dbSNP (https://www.ncbi.nlm.nih.gov/snp/) were excluded from the identified variants. Interpretation of the pathogenicity of somatic mutations was performed with reference to the Catalogue Of Somatic Mutations In Cancer (COSMIC, https://cancer.sanger.ac.uk/cosmic) and ClinVar (https://www.ncbi.nlm.nih.gov/clinvar/) databases.

### Statistical analyses

Data from IHC results were considered nominal variables. Correlations between IHC results and each histological component were analysed using the chi-square test and a statistical multivariate analysis method for nominal variables, a type III quantification method. *P* values of less than 0.05 were considered statistically significant. Correlation between IHC markers was analysed by Spearman correlation coefficient analysis. Statistical analyses were performed using BellCurve for Excel® (Social Survey Research Information Co., Ltd., Tokyo, Japan) and R version 4.2.0 (2022-04-22 ucrt), "Vigorous Calisthenics" Copyright (C) 2022 The R Foundation for Statistical Computing Platform: ×86_64-w64-mingw32/ × 64 (64-bit).

## Results

### Histopathological evaluation of components of MBC tumour masses

Clinicopathological data collected from the medical records for the twenty-five patients with MBC enrolled in this study are summarized (Supplementary Table 1). Results of pathological evaluation for MBC and/or non-MBC components consisting of each MBC tumour are shown in Supplementary Table 2. The most frequently detected MBC(c) was SCC(c) (n = 13), followed by SpCC(c) (n = 8), OSS/CAR(c) (n = 4), MPC(c) (n = 3), and PLM(c) (n = 4); the combination of these components differed among tumours (Supplementary Table 2). As non-MBC(c), normal(c) (n = 15), IC(c) (n = 9), and NST(c) (n = 13) were detected. A total of 69 components, including 32 MBC(c) and 37 non-MBC(c), were included for further examination in this study.

### Univariate analysis for the immunohistochemical characteristics of every histological component of MBC tumours

**Fig. 2:**
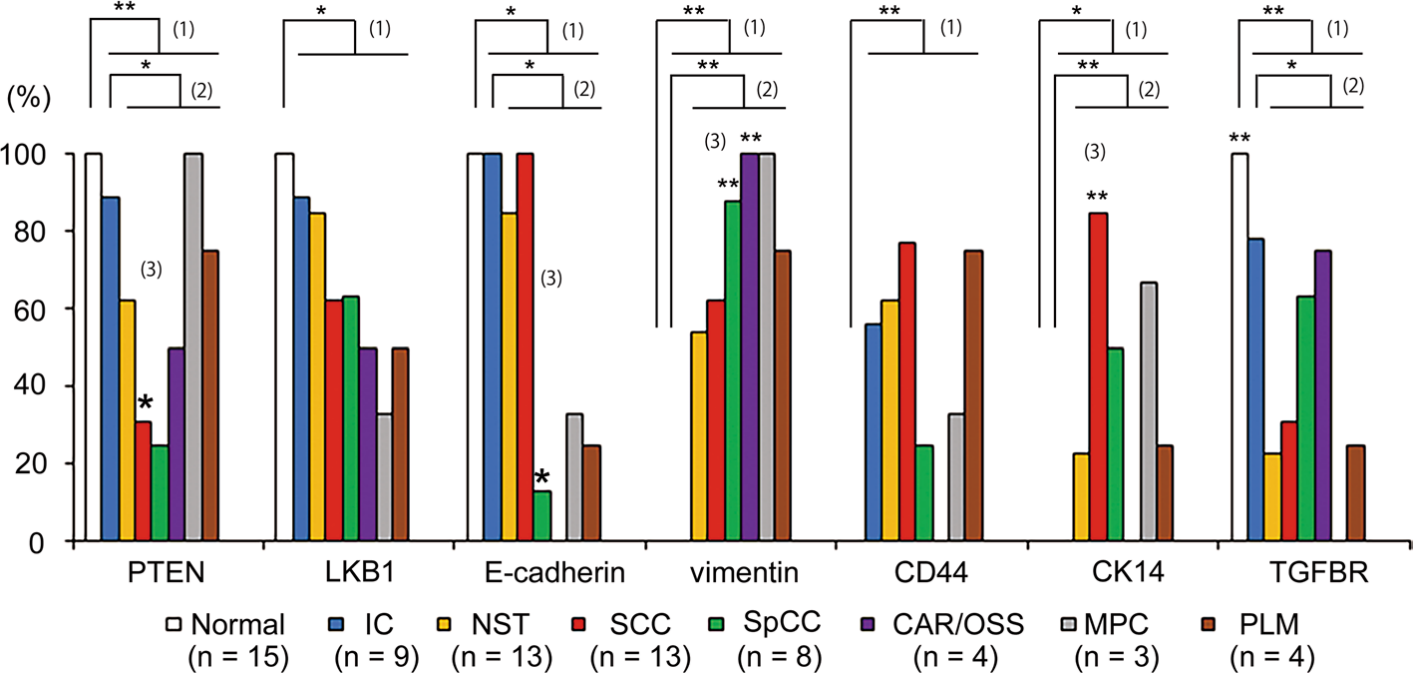
The positive percentage for seven markers in every component within MBC samples determined by immunohistochemistry. Comparisons were performed between (1) normal tissue (normal(c)) and carcinoma, (2) intraductal component (IC(c)) and invasive carcinoma, and (3) each metaplastic component (MBC(c)) and other components from all patients. White bars: normal tissue (normal(c)), blue bars: intraductal component (IC(c)), yellow bars: invasive ductal carcinoma of no special type (NST)(c), red bars: squamous cell carcinoma (SCC)(c), green bars: spindle cell carcinoma (SpCC)(c), purple bars: carcinoma with cartilaginous/osseous differentiation (CAR/OSS)(c), grey bars: matrix-producing carcinoma (MPC)(c), brown bars: pleomorphic carcinoma (PLM)(c). Percentages were calculated as the positive number/total number of samples for each component. Significance was determined by the chi-square test, *P < 0.05 and **P < 0.01. n: total number of each sample

To examine how MBC develops heterogeneous phenotypic characteristics, we evaluated the immunohistochemical features of all 69 components of 25 tumours individually. The percentage of the positive number/total number of components for each SCC(c), SpCC(c), OSS/CAR(c), MPC(c), and PLM(c), NST(c), IC(c) and normal (c) were calculated for seven markers (Fig. 2). Compared to normal(c) (designated as (1) in Fig. 2), the positive percentage for MBC(c) was a significant decrease for PTEN, LKB1, E-cadherin, and TGFBR and a significant increase for vimentin, CD44, CK14, and TGFBR. Compared to IC(c) (designated as (2) in Fig. 2), both NST(c) and MBC(c) showed decreased positivity for PTEN, E-cadherin, and TGFBR and increased positivity for vimentin and CK14.

Focusing on each individual MBC(c) (designated as (3) in Fig. 2), we found that a significantly lower percentage of SCC(c) was positive for PTEN (4/13, *P* < 0.05; red bars in Fig. 2; Supplementary Table 5), with a higher percentage for CK14 (11/13, *P* < 0.01), compared to other MBC(c)s. A significantly lower percentage of SpCC(c) was positive for E-cadherin (1/8, *P* < 0.05; green bars in Fig. 2) and a higher percentage was positive for vimentin (7/8, *p* < 0.05) compared to other MBC(c)s. In addition, CAR/OSS(c) showed a higher positive percentage for vimentin (purple bars in Fig. 2) (4/4, *p* < 0.05). Thus, SCC(c) and SpCC(c) showed unique immunohistochemical findings compared to other MBC components; SCC(c) was PTEN-negative, CK14-positive (basal-like phenotype) and SpCC(c) was E-cadherin-negative and vimentin-positive (epithelial mesenchymal transition (EMT)-like phenotype).

### Multivariate analysis for the immunohistochemical profile of every histological component of MBC tumours

**Fig. 3:**
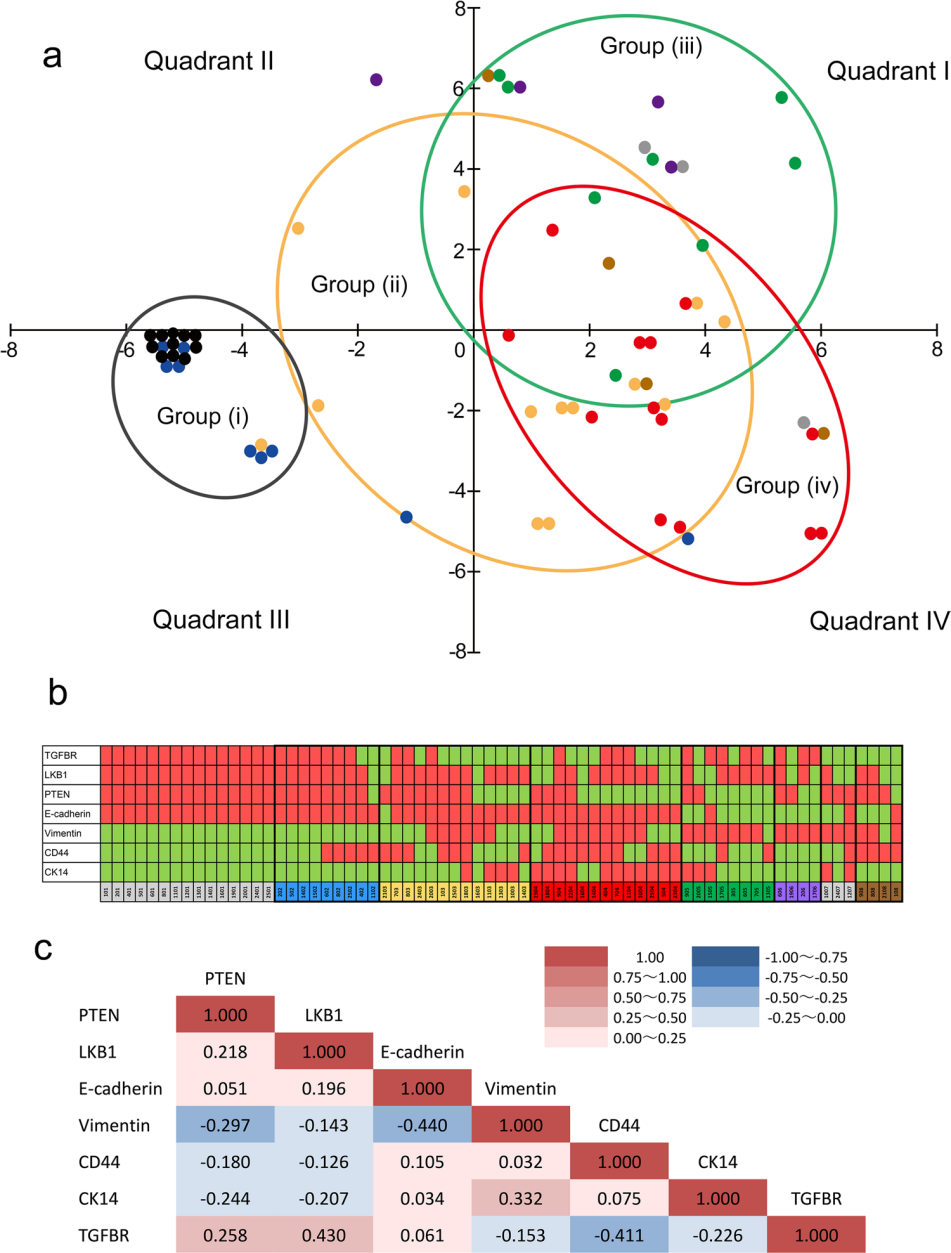
Immunohistochemical profiles of every component of metaplastic carcinoma. **a** Scatter diagram of the immunohistochemical expression pattern in different MBC components estimated by a type III quantification method. A type III quantification method is one of the standard methods for multivariate analysis of category variables. Quadrant I (right upper) is divided by the X-axis (PTEN negative, CK14 positive, LKB1 negative) and Y-axis (E-cadherin negative, vimentin positive, CD44 negative); quadrant II (left upper) is divided by the X-axis (PTEN positive, vimentin negative, TGFBR positive) and Y-axis (E-cadherin negative, vimentin positive, CD44 negative); quadrant III (left lower) is divided by the X-axis (PTEN positive, vimentin negative, TGFBR positive) and Y-axis (CD44 positive, vimentin negative, TGFBR negative); and quadrant IV (right lower) is divided by the X-axis (PTEN negative, CK14 positive, LKB1 negative) and Y-axis (CD44 positive, vimentin negative, TGFBR negative). Black dots, normal(c); blue dots, intraductal component (IC)(c); yellow dots, invasive ductal carcinoma of no special type (NST)(c); red dots, squamous cell carcinoma (SCC)(c); green dots, spindle cell carcinoma (SpCC)(c); purple dots, carcinoma with cartilaginous/osseous differentiation (CAR/OSS)(c); grey dots, matrix-producing carcinoma (MPC)(c); brown dots, pleomorphic carcinoma (PLM)(c). Group (i), circled by a black line; Group (ii), circled by a yellow line; Group (iii), circled by a green line; Group (iv), circled by a red line. **b** A panel presenting all of the positive or negative results by immunohistochemistry. Immunohistochemical results for antibodies are shown as red cells for positive, and green cell for negative. Dark grey cells, normal(c); blue cells, intraductal component (IC)(c); yellow cells, invasive ductal carcinoma of no special type (NST)(c); red cells, squamous cell carcinoma (SCC)(c); green cell, spindle cell carcinoma (SpCC)(c); purple cells, carcinoma with cartilaginous/osseous differentiation (CAR/OSS)(c); light grey cells, matrix-producing carcinoma (MPC)(c); brown cells, pleomorphic carcinoma (PLM)(c). **c**. Correlation between immunohistochemical expression of the markers used in this study analysed by Spearman’s rank correlation coefficient. Spearman’s correlation coefficients are shown in each corresponding cell. Red cells, positive correlation; blue cells, negative correlation.

In this study, we used multiple antibodies for multiple components, which may or may not correlate with each other. Therefore, we next evaluated the correlation between antibodies and components by a statistical multivariate analysis method for nominal variables, a type III quantification method. Using this approach, four groups were identified (Fig. 3a): Group (i), including normal(c) and IC(c), was plotted mainly on the minus side of the X-axis; Group (ii), including NST(c) samples, was distributed among all quadrants; Group (iii), including SpCC(c) samples, was plotted mainly in quadrant I; and Group (iv), including SCC(c) samples, was plotted predominantly in quadrant IV and partially in quadrant I. These results clearly showed that the IHC expression profiles of normal(c) and/or IC(c), NST(c), and MBC(c) were differently distributed, and furthermore, SCC(c) and SpCC(c) showed distinct distributions (Fig. 3a).

We next explored how these immunohistochemical markers are related using Spearman correlation coefficients; PTEN showed positive correlations with LKB1 and TGFBR, and negative correlations with vimentin, CK14, and CD44 (Fig. 3c). E-cadherin correlated negatively with vimentin.

Overall, multivariate analysis supported the results by univariate analysis that SCC(c), PTEN-negative, CK14-positive (basal-like phenotype) and SpCC(c), E-cadherin-negative and vimentin-positive (epithelial mesenchymal transition (EMT)-like phenotype) showed different IHC profiles.

### PTEN gene mutations detected in SCC(c)

Our immunohistochemical study demonstrated lower PTEN positivity for both SCC(c) and SpCC(c), though their IHC profiles differed. Therefore, we next sought to examine *PTEN* gene mutations as a possible cause of decreased PTEN protein expression using 12 available FFPE whole slides of MBC tumours with good preserved DNA quality. Two of 7 tumours containing SCC(c) showed *PTEN* gene mutations. In one patient (serial case no. 14), two missense mutations and two silent mutations were detected; a single deletion was detected in another SCC(c) sample (serial case no. 25) (Table 1). These detected *PTEN* gene mutations are shown in Supplementary Fig. 1. The samples from the remaining 5 patients containing SpCC(c), CAR/OSS(c), MPC(c) or PLM(c) exhibited a wild-type *PTEN* gene. Thus, *PTEN* mutations were detected only in the tumour containing SCC(c).

**Table 1:** Results of *PTEN* mutation analysis

Serial case no	MBC(c)	PTEN IHC	*PTEN* mutations	Amino acid changes	Function	Interpretation
14	SCC	−	c.249C > T c.265C > T c.453C > T c.976G > C	p.C83C p.P89S p.A151A p.D326N	Silent Missense Silent Missense	Unknown Unknown Unknown Unknown
25	SCC	−	c.955_958delACTT	p.Leu318_Thr319insTer	Stop gained	Pathogenic
16	SCC	−	WT	WT		
18	SCC	+	WT	WT		
22	SCC	+	WT	WT		
23	SCC	−	WT	WT		
19	SCC, CAR/OSS	+	WT	WT		
15	SpCC	−	WT	WT		
20	SpCC	+	WT	WT		
17	SpCC, CAR/OSS	−	WT	WT		
24	MPC	+	WT	WT		
21	PLM	+	WT	WT		

WT: wild type

### Next-generation sequencing analysis of MBC tumours containing SCC(c)

We next analysed the MBC components that gained gene alterations to evaluate the phenotypic and genetic correlation by NGS. Two candidate MBC tumours were from serial case no. 14 and no. 25, both of which were PTEN negative by IHC but positive for *PTEN* gene alterations (Table 1). However, the DNA quantity of the FFPE samples from case no. 14 was not sufficient for NGS analysis. Hence, we used case no. 16 in addition to case no. 25 (Table 2 and Supplementary Fig. 2).

**Table 2:** Results of next-generation sequencing analysis of 2 cases of MBC including SCC(c)

Serial case no	Gene	Chr	Locus	RefSeq	HGVS.c	HGVS.p	Function	Interpretation	VAF (%)		
									IC (c)	NST (c)	SCC (c)
16	*KIT*	4	55593464	NM_000222.2	c.1621A > C	p.Met541Leu	Missense	Likely benign	NT	47.8	52.1
	*KIT*	4	55602765	NM_000222.2	c.2586G > C	p.Leu862 =	Silent			52.8	49.9
	*IL7R*	5	35874575	NM_002185.5	c.731C > T	p.Thr244Ile	Missense	Benign		34.6	48.5
	*NOTCH1*	9	139397,707	NM_017617.5	c.5094C > T	p.Asp1698 =	Silent			28.8	50.6
	*PTEN*	10	89720852	NM_000314.4	c.1003C > T	p.Arg335Ter	Nonsense	Pathogenic		–	4.4
	*FLT3*	13	28608459	NM_004119.3	c.1683A > G	p.Leu561 =	Silent			44.5	45.7
	*RB1*	13	48942685	NM_000321.2	c.1072C > T	p.Arg358Ter	Nonsense	Pathogenic		–	4.3
	*TP53*	17	7577022	NM_000546.5	c.916C > T	p.Arg306Ter	Nonsense	Pathogenic		19.5	25.8
25	*MSH2*	2	47703500	NM_000251.2	c.2006-6 T > C		Intronic	Benign	50.9	48.6	51.4
	*PIK3CA*	3	178952085	NM_006218.4	c.3140A > G	p.His1047Leu	Missense	Pathogenic	7	36.6	32.1
	*IL7R*	5	35874575	NM_002185.5	c.731C > T	p.Thr244Ile	Missense	Benign	48.6	52.4	45
	*NOTCH1*	9	139397707	NM_017617.5	c.5094C > T	p.Asp1698 =	Silent		51.6	49.1	47.5
	*PTEN*	10	89720799	NM_000314.8	c.955_958del	p.Leu318_Thr319insTer	Stop gained	Pathogenic	–	14.7	21.9

Chr: chromosome; RefSeq: NCBI accession number; HGVS: human genome variant society; VAF: variant allele frequency; NT: not tested; –: not detected; IC: intraductal component; NST: no special type; SCC: squamous cell carcinoma

For case no. 16, eight somatic gene mutations were identified, which were contained in both NST(c) and SCC(c). Specifically, two mutations were identified in *KIT,* and one mutation was identified in each of the *IL7R*, *NOTCH*, *PTEN*, *FLT3*, *RB1* and *TP53* genes (Table 2). The mutations identified in *PTEN*, *RB1* and *TP53* are nonsense variants that have been reported as pathogenic in COSMIC database. Within this tissue sample, the pathogenic *TP53* mutation was detected at a lower VAF in NST(c) (19.5%) than in SCC(c) (25.8%). Additionally, pathogenic *PTEN* and *RB1* mutations were detected in SCC(c) at relatively low VAFs of 4.4% and 4.3%, respectively, but were not detected in NST(c).

For case no. 25, five somatic gene mutations were detected in IC(c), NST(c) and SCC(c) (Supplementary Fig. 2). Specifically, mutations in *MSH2*, *PIK3CA*, *IL7R*, *NOTCH* and *PTEN* were identified*,* and those in *PIK3CA* and *PTEN* were confirmed as pathogenic variants in the COSMIC database. The pathogenic *PIK3CA* mutation was detected in IC(c) at a relatively low VAF (7%), with a higher VAF (36.6% and 32.1%) detected in NST(c) and SCC(c), respectively. The pathogenic *PTEN* mutation was not detected in IC(c) but was detected in NST(c) and SCC(c), at VAFs of 14.7% and 21.9%, respectively.

## Discussion

The present study demonstrated that MBC tumour masses most frequently consisted of SCC(c) and SpCC(c). SCC(c) were significantly PTEN negative and CK14 positive (basal-like phenotype), and SpCC(c) were significantly E-cadherin negative and vimentin positive (EMT-like phenotype). Multivariate analyses showed the immunohistochemical profiles of IC(c), NST(c), and MBC(c) to be distributed differently, and moreover, SCC(c) and SpCC(c) distinctly grouped. *PTEN* gene mutations were detected only in SCC(c), but not in SpCC(c). Thus, SCC(c) and SpCC(c) presented different immunohistochemical and molecular phenotypes. Based on NGS analyses, *PTEN* gene mutation was increasingly detected from IC(c) to NST(c) to SCC(c).

There are several lines of evidence showing that PTEN loss contributes to a squamous phenotype. Mouse models addressing the contribution of PTEN loss to SCC in non-mammary organs have been reported. One of such studies for lung SCC showed that biallelic inactivation of *Lkb1* and *PTEN* is required to induce squamous differentiation [16]. In addition, Squarize et al. [17] reported that a mouse model of Cowden syndrome, a syndrome associated with germline mutations in *PTEN* [18], presents a phenotype of acral keratosis that is reversed by induced *PTEN* expression [17]. Another mouse model induced by *PTEN* deletion shows progression of epidermal hyperplasia, hypergranulosis, hyperkeratosis, mammary adenosquamous carcinoma, adenomyoepithelioma [19, 20], and cutaneous SCC, accompanied by hyperactivation of mTOR and production of fibroblast growth factor (FGF) [20].

In terms of studies on human cancer tissue, *PTEN* gene mutations are more frequently detected in SCC than in adenocarcinoma, e.g., 19% of lung [21], 15% of head and neck [22], 11% of oesophageal [23], and 6%–11% of other cancers [15, 24]. For breast carcinoma, PTEN loss is observed in 40–50% of cases, and dysfunction of PTEN with frameshift mutations and epigenetic mechanisms is detected in 5–10% [19]. Another study of MBC showed that *PTEN* gene mutations were more frequently detected in SCC (3/9, 33%) than in SpCC (0/10, 0%) and CAR (1/16, 6%) [10]. The relationship between SCC(c) and PTEN loss has thus been demonstrated considering the entire SCC tumour mass. The present study is the first to reveal that PTEN loss and *PTEN* gene mutations are characteristically detected in SCC(c).

One of the unaddressed questions for MBC is whether its heterogeneous phenotypes develop during tumour progression via invasive carcinoma or occur through a de novo mechanism. The interesting findings of this study by NGS analysis were as follows: (1) gene mutations were highly shared between NST(c) and SCC(c) in case no. 16 (6 of 8 genes) and between IC(c), NSTC(c) and SCC(c) in case no. 25 (4 of 5 genes); (2) pathologic mutations in *PTEN, RB1* and *TP53* were increasingly detected with higher VAF in SCC(c) than in NST(c) in case no. 16. In case no. 25, no *PTEN* gene mutation was detected in IC(c) but was increasingly detected in NST(c) and SCC(c). These findings suggest that SCC(c) occurs via NST(c) rather than de novo from non-neoplastic epithelial cells. Considering all the findings together, SCC(c) might develop via NST(c), but not de novo, gaining *PTEN* gene mutation that affected the SCC(c) phenotype, which process was distinctive from that of SpCC(c).

Possible causes of the decreased PTEN positivity observed in SpCC(c), independent of genetic alterations, are an EMT mechanism, *PTEN* gene promoter methylation [25], and miRNA involvement [26]. *PTEN* promoter methylation is present in 31.1% of breast carcinoma cases, 64.3% of involve loss of PTEN expression [25]. If the pathogenesis of SCC(c) and SpCC(c) is based on different mechanisms, appropriate adjuvant chemotherapeutic strategies would also differ. More effective therapies for each component, such as AKT signaling inhibitors for SCC and EMT inhibitors for SpCC, may be proposed.

A major limitation of this study is its small sample size. The relatively low incidence of MBC makes it difficult to collect sufficient samples with good-quality DNA for molecular analysis over a short duration, because longer duration of formalin fixation increases artefactual modifications in DNA [27], and longer storage of FFPE samples increases fragmentation of DNA strands [28, 29]. A related issue is that we could not analyse CAR/OSS, MPC or PLM samples in this study cohort. One potential solution for studying tumours with low incidence over a short duration, with good fixation and storage conditions, is to perform a multi-institutional study, and this approach will be considered in the future.

## Conclusion

Although SCC(c) and SpCC(c) are occasionally admixed in a single tumour, these components showed distinct immunohistochemical profiles: SCC(c) and SpCC(c) were grouped in the basal phenotype and in the EMT-like phenotype, respectively. Negative expression of *PTEN* in SCC(c) was accompanied by *PTEN* gene mutation but not in SpCC(c). NGS analyses demonstrated *PTEN* gene mutation to be progressively increased from IC(c) to NST(c) to SCC(c). Immunohistochemical and molecular profiles of SCC(c) were distinct from those of SpCC(c) and may originate through NST(c).

### Supplementary Information

Below is the link to the electronic supplementary material

Supplementary file1 (DOCX 47 KB)

Supplementary file2 (TIF 45626 KB)

Supplementary file3 (TIF 8684 KB)

## Data Availability

The datasets generated during and/or analysed during the current study are available from the corresponding author on reasonable request.

## References

[1] Bosman FT, World Health Organization (2019). International Agency for Research on Cancer. WHO classification of tumours. Breast Tumour.

[2] Lee H, Jung SY, Ro JY, Kwon Y, Sohn JH, Park IH, Lee KS, Lee S, Kim SW, Kang HS, Ko KL, Ro J (2012). Metaplastic breast cancer: clinicopathological features and its prognosis. J Clin Pathol.

[3] Rakha EA, Tan PH, Varga Z, Tse GM, Shaaban AM, Climent F, Van Deurzen CH, Purnell D, Dodwell D, Chan T, Ellis IO (2015). Prognostic factors in metaplastic carcinoma of the breast: a multi-institutional study. Br J Cancer.

[4] Song Y, Liu X, Zhang G (2013). Unique clinicopathological features of metaplastic breast carcinoma compared with invasive ductal carcinoma and poor prognostic indicators. World J Surg Oncol.

[5] Yamaguchi R, Horii R, Maeda I, Suga S, Makita M, Iwase T, Oguchi M, Ito Y, Akiyama F (2010). Clinicopathologic study of 53 metaplastic breast carcinomas: their elements and prognostic implications. Hum Pathol.

[6] Weigelt B, Kreike B, Reis-Filho JS (2009). Metaplastic breast carcinomas are basal-like breast cancers: a genomic profiling analysis. Breast Cancer Res Treat.

[7] Weigelt B, Ng CK, Shen R, Popova T, Schizas M, Natrajan R, Mariani O, Stern MH, Norton L, Vincent-Salomon A, Reis-Filho JS (2015). Metaplastic breast carcinomas display genomic and transcriptomic heterogeneity [corrected]. Mod Pathol.

[8] Edenfield J, Schammel C, Collins J, Schammel D, Edenfield WJ (2017). Metaplastic breast cancer: molecular typing and identification of potential targeted therapies at a single institution. Clin Breast Cancer.

[9] Reed AEM, Kalaw E, Nones K (2019). Phenotypic and molecular dissection of metaplastic breast cancer and the prognostic implications. J Pathol.

[10] Ng CKY, Piscuoglio S, Geyer FC (2017). The landscape of somatic genetic alterations in metaplastic breast carcinomas. Clin Cancer Res.

[11] Ross JS, Badve S, Wang K (2015). Genomic profiling of advanced-stage, metaplastic breast carcinoma by next-generation sequencing reveals frequent, targetable genomic abnormalities and potential new treatment options. Arch Pathol Lab Med.

[12] Zhai J, Giannini G, Ewalt MD, Zhang EY, Invernizzi M, Niland J, Lai LL (2019). Molecular characterization of metaplastic breast carcinoma via next-generation sequencing. Hum Pathol.

[13] Kobayashi H, Nakai T, Nakanishi Y, Esumi M, Masuda S (2021). Phylogenetic analysis of combined lobular and ductal carcinoma of the breast. Mol Med Rep.

[14] Reed AEM, Kutasovic JR, Nones K (2018). Mixed ductal-lobular carcinomas: evidence for progression from ductal to lobular morphology. J Pathol.

[15] Ali SM, Yao M, Yao J (2017). Comprehensive genomic profiling of different subtypes of nasopharyngeal carcinoma reveals similarities and differences to guide targeted therapy. Cancer.

[16] Xu C, Fillmore CM, Koyama S (2014). Loss of Lkb1 and Pten leads to lung squamous cell carcinoma with elevated PD-L1 expression. Cancer Cell.

[17] Squarize CH, Castilho RM, Gutkind JS (2008). Chemoprevention and treatment of experimental Cowden's disease by mTOR inhibition with rapamycin. Cancer Res.

[18] Banneau G, Guedj M, MacGrogan G (2010). Molecular apocrine differentiation is a common feature of breast cancer in patients with germline PTEN mutations. Breast Cancer Res.

[19] Carbognin L, Miglietta F, Paris I, Dieci MV (2019). Prognostic and predictive implications of PTEN in breast cancer: unfulfilled promises but intriguing perspectives. Cancers (Basel).

[20] Hertzler-Schaefer K, Mathew G, Somani AK, Tholpady S, Kadakia MP, Chen Y, Spandau DF, Zhang X (2014). Pten loss induces autocrine FGF signaling to promote skin tumorigenesis. Cell Rep.

[21] Lee HY, Lee SH, Won JK (2017). Analysis of fifty hotspot mutations of lung squamous cell carcinoma in never-smokers. J Korean Med Sci.

[22] Chung CH, Guthrie VB, Masica DL (2015). Genomic alterations in head and neck squamous cell carcinoma determined by cancer gene-targeted sequencing. Ann Oncol.

[23] Wang K, Johnson A, Ali SM (2015). Comprehensive genomic profiling of advanced esophageal squamous cell carcinomas and esophageal adenocarcinomas reveals similarities and differences. Oncologist.

[24] Ojesina AI, Lichtenstein L, Freeman SS (2014). Landscape of genomic alterations in cervical carcinomas. Nature.

[25] Zhang HY, Liang F, Jia ZL, Song ST, Jiang ZF (2013). PTEN mutation, methylation and expression in breast cancer patients. Oncol Lett.

[26] Han M, Liu M, Wang Y, Chen X, Xu J, Sun Y, Zhao L, Qu H, Fan Y, Wu C (2012). Antagonism of miR-21 reverses epithelial-mesenchymal transition and cancer stem cell phenotype through AKT/ERK1/2 inactivation by targeting PTEN. PLoS ONE.

[27] Do H, Dobrovic A (2015). Sequence artifacts in DNA from formalin-fixed tissues: causes and strategies for minimization. Clin Chem.

[28] Hatanaka Y, Kuwata T, Morii E (2021). The Japanese Society of Pathology Practical Guidelines on the handling of pathological tissue samples for cancer genomic medicine. Pathol Int.

[29] Kanai Y, Nishihara H, Miyagi Y (2018). The Japanese Society of Pathology Guidelines on the handling of pathological tissue samples for genomic research: standard operating procedures based on empirical analyses. Pathol Int.

