# Supplementary material for: Immunohistochemical and molecular profiles of heterogeneous components of metaplastic breast cancer: a squamous cell carcinomatous component was distinct from a spindle cell carcinomatous component
Source: Discov Oncol. 2024 Apr 2;15:95. doi: 10.1007/s12672-024-00950-0 (PMC10987432; doi:10.1007/s12672-024-00950-0)
Supplement: Supplementary file 1 — Supplementary file1 (DOCX 47 KB) [file 12672_2024_950_MOESM1_ESM.docx]

**Supplementary Information**

**Supplementary Table 1**. Clinicopathological characteristics of patients with MBC tumours enrolled in this study

| **Factors** | **MBC patients (n = 25)** |
| --- | --- |
| Age, mean (range) | 62.16 (41–82) |
| Tumour size (cm) |  |
| <2 | 10 (40%) |
| 2–5 | 9 (36%) |
| >5 | 6 (24%) |
| Nuclear grade |  |
| 1 | 3 (12%) |
| 2 | 8 (32%) |
| 3 | 14 (56%) |
| Stage |  |
| I | 9 (36%) |
| II | 13 (52%) |
| III | 2 (8%) |
| IV | 1 (4%) |
| Lymph vessel invasion |  |
| Negative | 18 (72%) |
| Positive | 7 (28%) |
| Blood vessel invasion |  |
| Negative | 21 (84%) |
| Positive | 4 (16%) |
| Lymph node metastasis |  |
| Negative | 21 (84%) |
| Positive | 4 (16%) |
| Distant metastasis |  |
| Negative | 24 (96%) |
| Positive | 1 (4%) |
| Recurrence |  |
| Negative | 23 (92%) |
| Positive | 2 (8%) |
| Subtype |  |
| Luminal | 3 (12%) |
| Her2 | 1 (4%) |
| Triple negative | 21 (84%) |

The presented data are the number of patients (percentage of total).

MBC, metaplastic breast carcinoma; Statistical analyses: Mann‒Whitney U test; all others, chi-square test ***P* < 0.01

**Supplementary Table 2**. Clinicopathological features of MBC in our patient cohort

| Data collected from medical records | | | | | | | |  | Histopathological evaluation in this study | | | | | | | | |
| --- | --- | --- | --- | --- | --- | --- | --- | --- | --- | --- | --- | --- | --- | --- | --- | --- | --- |
| Serial case no. | Age | pStage | TNM | | | Recurrence | Pathological diagnosis |  | MBC component | | | | |  | Non-MBC component | | |
|  |  |  | pT | pN | cM |  |  |  | SCC | SpCC | CAR/  OSS | MPC | PLM |  | NST | IC | normal |
| 4 | 74 | IIIA | 3 | + | - | - | SCC |  | + |  |  |  |  |  |  | + | + |
| 5 | 71 | IIA | 1 | + | - | - | Mixed |  | + |  |  |  |  |  |  | + | + |
| 11 | 41 | IIA | 2 | - | - | - | Mixed |  | + |  |  |  |  |  | + | + | + |
| 14 | 60 | I | 1 | - | - | - | Mixed |  | + |  |  |  |  |  | + | + | + |
| 16 | 55 | I | 1 | - | - | - | Mixed |  | + |  |  |  |  |  | + |  | + |
| 18 | 51 | I | 1 | - | - | - | Mixed |  | + |  |  |  |  |  | + |  |  |
| 22 | 70 | IIA | 2 | - | - | - | SCC |  | + |  |  |  |  |  |  |  |  |
| 23 | 46 | IIA | 2 | - | - | - | SpCC |  | + |  |  |  |  |  |  |  |  |
| 25 | 67 | IIB | 3 | - | - | - | Mixed |  | + |  |  |  |  |  | + | + | + |
| 10 | 82 | IV | 4b | + | + | - | Mixed |  | + |  |  | + |  |  | + |  |  |
| 19 | 72 | I | 1 | - | - | - | Mixed |  | + |  | + |  |  |  |  |  | + |
| 7 | 65 | IIA | 2 | - | - | - | Mixed |  | + | + |  |  |  |  | + |  |  |
| 9 | 55 | IIA | 2 | - | - | - | Mixed |  | + | + |  |  | + |  |  |  |  |
| 3 | 53 | IIIC | 4b | + | - | - | Mixed |  |  | + |  |  |  |  |  |  |  |
| 13 | 55 | IIA | 2 | - | - | + | SpCC |  |  | + |  |  |  |  | + |  | + |
| 15 | 80 | I | 1 | - | - | - | SpCC |  |  | + |  |  |  |  |  | + |  |
| 20 | 78 | I | 1 | - | - | - | SpCC |  |  | + |  |  |  |  | + |  | + |
| 6 | 54 | IIA | 2 | - | - | - | CAR/OSS |  |  | + | + |  |  |  |  | + | + |
| 17 | 66 | IIB | 3 | - | - | - | CAR/OSS |  |  | + | + |  |  |  |  |  |  |
| 2 | 51 | I | 1 | - | - | - | Mixed |  |  |  | + |  |  |  |  | + | + |
| 12 | 62 | I | 1 | - | - | + | Mixed |  |  |  |  | + |  |  |  |  | + |
| 24 | 44 | IIA | 2 | - | - | - | Mixed |  |  |  |  | + |  |  | + |  | + |
| 1 | 57 | IIA | 2 | - | - | - | SpCC |  |  |  |  |  | + |  | + |  | + |
| 8 | 69 | I | 1 | - | - | - | PLM |  |  |  |  |  | + |  | + | + | + |
| 21 | 76 | IIB | 3 | - | - | - | PLM |  |  |  |  |  | + |  | + |  |  |

Lines in this table are ordered according to the results of the histopathological evaluation in this study (right columns). The order is primarily the frequency of MBC(c), SCC(c), SpCC(c), CAR/OCC(c), MPC(c), and PLMC(c) and secondarily normal(c), IC(c) and NST(c).

pT, T categories of TNM pathological classification; LN, lymph node; MBC, metaplastic breast carcinoma; SCC, squamous cell carcinoma; SpCC, spindle cell carcinoma; CAR/OSS, carcinoma with cartilaginous/osseous differentiation; MPC, matrix-producing carcinoma; PLM, pleomorphic carcinoma; NST, invasive ductal carcinoma of no special type; IC, intraductal component; Mixed, mixed NST and MBC

**Supplementary Table 3.** Primary antibodies used for immunohistochemistry

| **Antibody target** | **Animal** | **Clone** | **Dilution** | **Antigen retrieval method** | **Company** |
| --- | --- | --- | --- | --- | --- |
| E-cadherin | Mouse | NCH-38 | 1:50 | TRS, pH 9.0, boil | Agilent, Santa Clara, CA, USA |
| Vimentin | Mouse | V9 | 1:200 | Citric buffer, pH 6.0, boil | Agilent, Santa Clara, CA, USA |
| PTEN | Rabbit | EPR9941-2 | 1:50 | TRS, pH 9.0, boil | Abcam, Cambridge, UK |
| LKB1 | Rabbit | D60C5F10 | 1:250 | Citric buffer, pH 6.0, boil | Cell Signaling Technology, Inc., Danvers, MA, USA |
| p40 | Rabbit | - | 1:2 | TRS, pH 9.0, boil | Nichirei Bioscience, Inc., Tokyo, Japan |
| CK14 | Mouse | LL002 | 1:100 | Citric buffer, pH 6.0, boil | Leica Biosystems, Nussloch, Germany |
| TGFBR1 | Rabbit | - | 1:2000 | Citric buffer, pH 6.0, boil | Cusabio Technology, Houston, TX, USA |
| CD44 | Mouse | DF1485 | 1:300 | Citric buffer, pH 6.0, boil | Leica Biosystems, Nussloch, Germany |

**Supplementary Table 4.** Primers used for *PTEN* gene mutation assays

| **Target** | **Accession number (NCBI)** | **Forward primer** | **Tm (°C)** | **Reverse primer** | **Tm (°C)** | **Product size (bp)** |
| --- | --- | --- | --- | --- | --- | --- |
| Exon 1 | AF067844.1 | 5′-tccatcctgcagaagaagcc-3′ | 59.7 | 5′-tccgtctactcccacgttct-3′ | 60 | 227 |
| Exon 2 | AF067844.1 | 5′-acttcttttagtttgattgctgca-3′ | 57.9 | 5′-tttctgtggcttagaaatcttttct-3′ | 57.2 | 234 |
| Exon 3 | AF067844.1 | 5′-tctgtcttttggtttttcttgatagt-3′ | 57.5 | 5′-actctacctcactctaacaagca-3′ | 58.6 | 231 |
| Exon 4 | AF067844.1 | 5′-aagattcaggcaatgtttgttagt-3′ | 57.3 | 5′-tcactcgataatctggatgactca-3′ | 58.9 | 232 |
| Exon 5-1 | AF067844.1 | 5′-tgcaacatttctaaagttacctacttg-3′ | 58.4 | 5′-cagttcgtccctttccagct-3′ | 60 | 225 |
| Exon 5-2 | AF067844.1 | 5′-tgtgaagatcttgaccaatggc-3′ | 58.9 | 5′-cagatccaggaagaggaaagga-3′ | 58.9 | 224 |
| Exon 6-1 | AF067844.1 | 5′-acccagttaccatagcaatttagtg-3′ | 59.1 | 5′-tccgccactgaacattggaa-3′ | 59.9 | 220 |
| Exon 6-2 | AF067844.1 | 5′-tccaccagggagtaactattcc-3′ | 58.6 | 5′-tggttaagaaaactgttccaataca-3′ | 57.1 | 203 |
| Exon 7-1 | AF067844.1 | 5′-tgacagtttgacagttaaaggca-3′ | 58.7 | 5′-agcatcttgttctgtttgtgga-3′ | 58.4 | 223 |
| Exon 7-2 | AF067844.1 | 5′-cacacgacgggaagacaagt-3′ | 60 | 5′-agcaaaacacctgcagatct-3′ | 57.7 | 244 |
| Exon 8-1 | AF067844.1 | 5′-tgcaaatgtttaacataggtgacaga-3′ | 59.5 | 5′-tctgcacgctctatactgca-3′ | 58.9 | 235 |
| Exon 8-2 | AF067844.1 | 5′-accaggaccagaggaaacct-3′ | 60.1 | 5′-agtcaccaacccccacaaaa-3′ | 59.7 | 224 |
| Exon 9-1 | AF067844.1 | 5′-tgttcatctgcaaaatggaataaaaa-3′ | 57.1 | 5′-ggatcagagtcagtggtgtca-3′ | 59.1 | 240 |
| Exon 9-2 | AF067844.1 | 5′-gccgtcaaatccagaggcta-3′ | 59.8 | 5′-aggtccattttcagtttattcaagt-3′ | 57.2 | 219 |

**Supplementary Table 5.** Immunohistochemical results for each histologically identified component of MBC

| component | **n** | **PTEN** | **LKB1** | **E-cadherin** | **Vimentin** | **CD44** | **CK14** | **TGFBR** |
| --- | --- | --- | --- | --- | --- | --- | --- | --- |
| Normal | 15 | 15 | 15 | 15 | **0**** | **0**** | **0*** | **15**** |
| IC | 9 | 8 | 8 | 9 | **0*** | 5 | 0 | 7 |
| NST | 13 | 8 | 11 | 11 | 7 | 8 | 3 | 3 |
| SCC | 13 | **4*** | 8 | 13 | 8 | 10 | **11**** | 4 |
| SpCC | 8 | 2 | 5 | **1*** | **7*** | 2 | 4 | 5 |
| CAR/OSS | 4 | 2 | 2 | 0 | **4*** | 0 | 0 | 3 |
| MPC | 3 | 3 | 1 | 1 | 3 | 1 | 2 | 0 |
| PLM | 4 | 3 | 2 | 1 | 3 | 3 | 1 | 1 |

**P <* 0.05, ***P* < 0.01 (chi-square test); n, number of MBC components

IC, intraductal component; NST, invasive ductal carcinoma of no special type; SCC, squamous cell carcinoma; SpCC, spindle cell carcinoma; CAR/OSS, carcinoma with cartilaginous/osseous differentiation; MPC, matrix-producing carcinoma; PLM, pleomorphic carcinoma

**Supplementary information**

**Supplementary Fig. 1** Results of *PTEN* gene sequencing

Gene alterations detected in case no. 14 and case no. 25 are shown. Two silent mutations, 2 missense mutations and 1 deletion are indicated (▼).

**Supplementary Fig. 2** Histological images of MBC tissues with SCC(c) subjected to next-generation sequencing analysis

Haematoxylin and eosin staining results for case no. 16 (a; NST(c) and b; SCC(c)) and case no. 25 (c; IC(c), d; NST(c) and e; SCC(c)) are presented. Scale bars: 100 µm (a, b, d, e), 50 µm (c)
