# Supplementary figures and images for: Immunohistochemical and molecular profiles of heterogeneous components of metaplastic breast cancer: a squamous cell carcinomatous component was distinct from a spindle cell carcinomatous component
Source: Discov Oncol. 2024 Apr 2;15:95. doi: 10.1007/s12672-024-00950-0 (PMC10987432; doi:10.1007/s12672-024-00950-0)

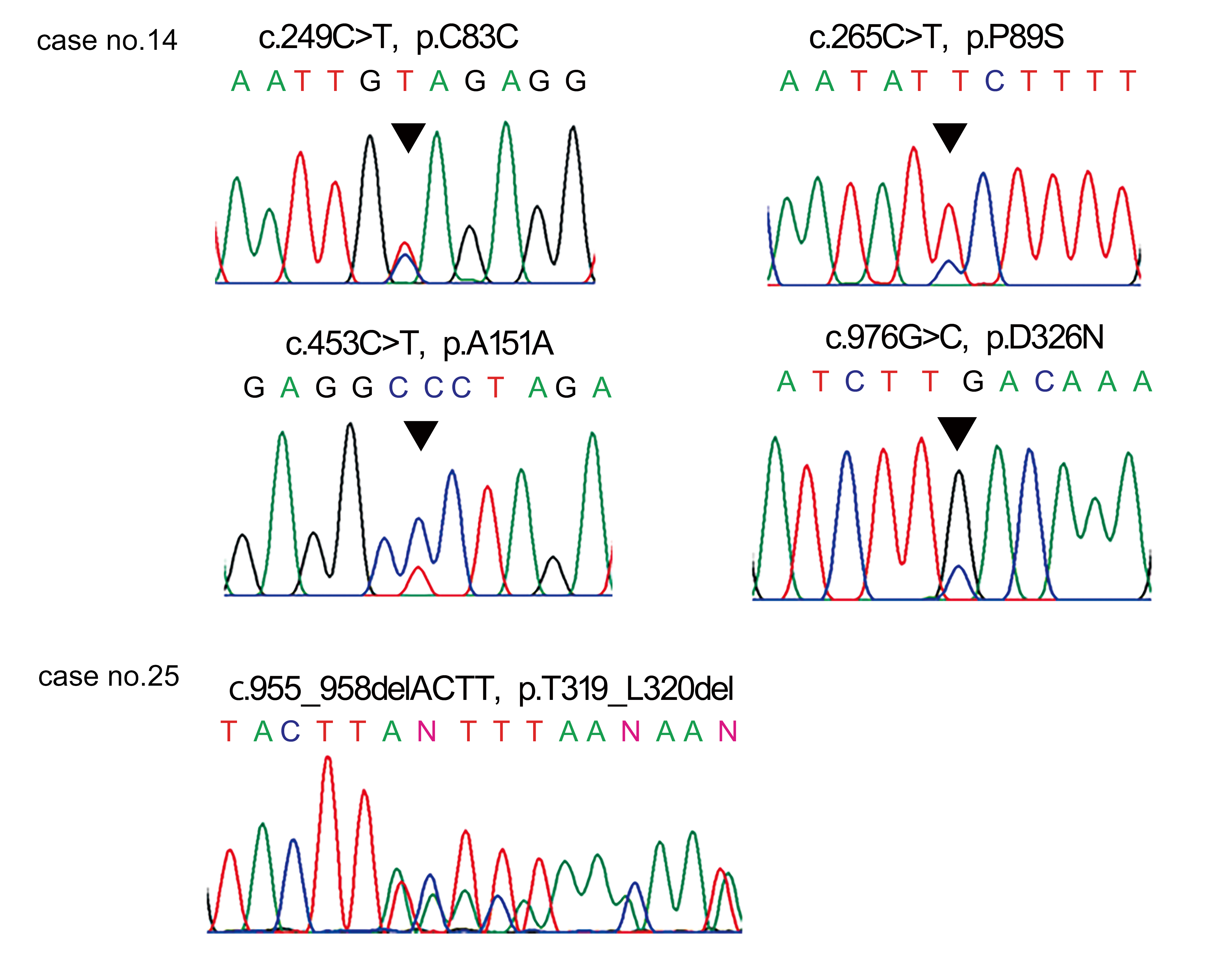

Supplement: Supplementary file 2 — Supplementary file2 (TIF 45626 KB) [file 12672_2024_950_MOESM2_ESM.tif]

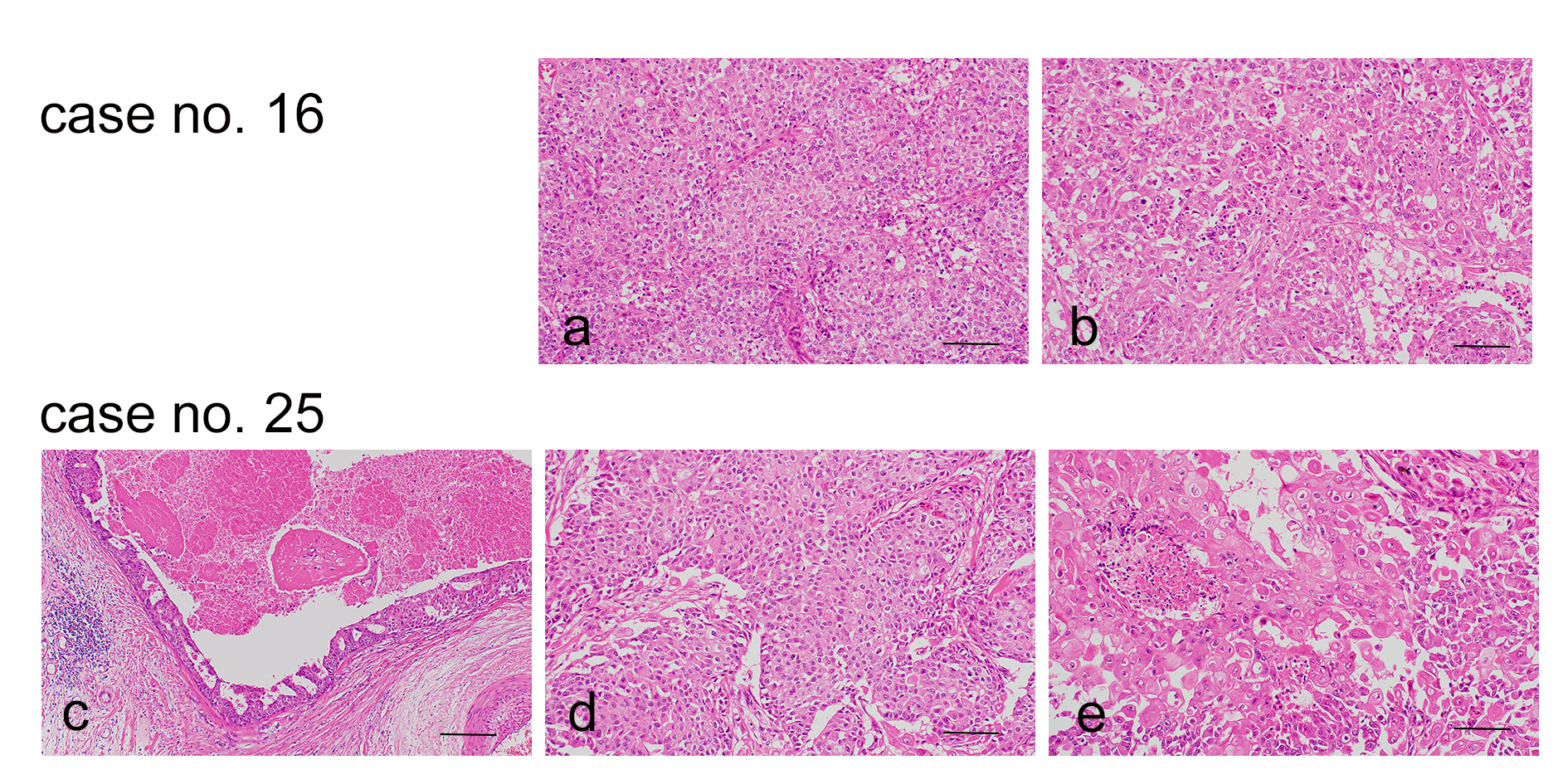

Supplement: Supplementary file 3 — Supplementary file3 (TIF 8684 KB) [file 12672_2024_950_MOESM3_ESM.tif]
